# Crystal structure of (2-methyl-4-phenyl-4*H*-benzo[4,5]thia­zolo[3,2-*a*]pyrimidin-3-yl)(phen­yl)methanone

**DOI:** 10.1107/S2056989015006428

**Published:** 2015-04-02

**Authors:** T. Sankar, S. Naveen, N. K. Lokanath, K. Gunasekaran

**Affiliations:** a Centre of Advanced Study in Crystallography and Biophysics, University of Madras, Guindy Campus, Chennai 600 025, India; b Institution of Excellence, University of Mysore, Manasagangotri, Mysore 570 006, India; c Department of Studies in Physics, University of Mysore, Manasagangotri, Mysore 570 006, India

**Keywords:** crystal structure, pyrimidine, benzo, thia­zolo, C—H⋯N hydrogen bonding

## Abstract

In the title compound, C_24_H_18_N_2_OS, the pyrimidine ring has a flat envelope conformation with the methine C atom as the flap. The attached phenyl and benzoyl rings are inclined to the mean plane of the pyrimidine ring by 84.87 (8) and 75.33 (9)°, respectively. The benzo­thia­zolo group is planar (r.m.s. deviation = 0.009 Å) and inclined to the mean plane of the pyrimidine ring by 3.27 (6)°. In the crystal, mol­ecules are linked by pairs of C—H⋯N hydrogen bonds, forming inversion dimers.

## Related literature   

For general background to the biological activities of pyrimidine derivatives, see: Kumar *et al.* (2002 ▸); Baraldi *et al.* (2002 ▸); Nasr & Gineinah (2002 ▸). For literature on the synthesis of fused benzo­thia­zolo derivatives, see: Nagarapu *et al.* (2013*a* ▸,*b* ▸).
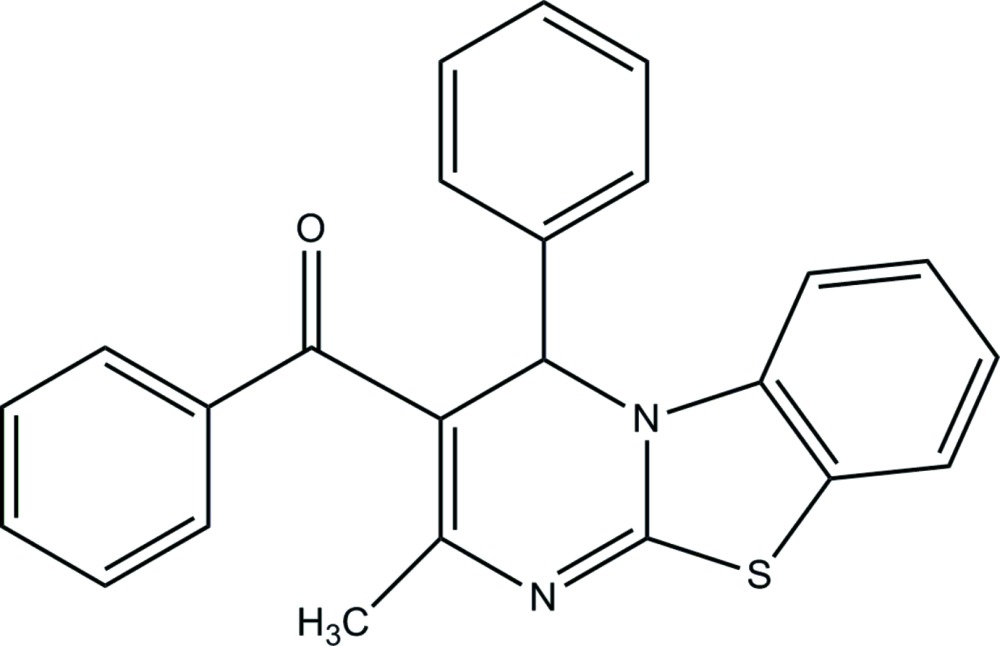


## Experimental   

### Crystal data  

C_24_H_18_N_2_OS
*M* *_r_* = 382.46
Monoclinic, 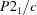

*a* = 12.1894 (6) Å
*b* = 18.6119 (8) Å
*c* = 8.9370 (4) Å
β = 110.360 (1)°
*V* = 1900.85 (15) Å^3^
*Z* = 4
Cu *K*α radiation
μ = 1.64 mm^−1^
*T* = 296 K
0.25 × 0.20 × 0.18 mm

### Data collection  

Bruker SMART APEXII CCD diffractometer
Absorption correction: multi-scan (*SADABS*; Bruker, 2008 ▸) *T*_min_ = 0.709, *T*_max_ = 0.745
11594 measured reflections
3090 independent reflections
3047 reflections with *I* > 2σ(*I*)
*R*_int_ = 0.036

### Refinement  

*R*[*F* ^2^ > 2σ(*F* ^2^)] = 0.040
*wR*(*F* ^2^) = 0.109
*S* = 1.08
3090 reflections
254 parameters
H-atom parameters constrained
Δρ_max_ = 0.28 e Å^−3^
Δρ_min_ = −0.33 e Å^−3^

### 

Data collection: *APEX2* (Bruker, 2008 ▸); cell refinement: *SAINT* (Bruker, 2008 ▸); data reduction: *SAINT*; program(s) used to solve structure: *SHELXS97* (Sheldrick, 2008 ▸); program(s) used to refine structure: *SHELXL97* (Sheldrick, 2008 ▸); molecular graphics: *ORTEP-3 for Windows* (Farrugia, 2012 ▸); software used to prepare material for publication: *SHELXL97* and *PLATON* (Spek, 2009 ▸).

## Supplementary Material

Crystal structure: contains datablock(s) global, I. DOI: 10.1107/S2056989015006428/su5105sup1.cif

Structure factors: contains datablock(s) I. DOI: 10.1107/S2056989015006428/su5105Isup2.hkl

Supporting information file. DOI: 10.1107/S2056989015006428/su5105Isup3.cml

**Fig. 1.**
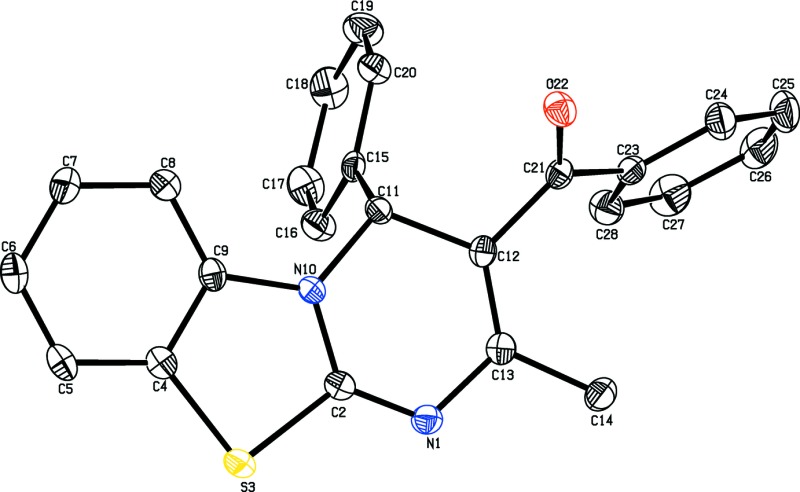
The molecular structure of the title compound, with atom labelling. The displacement ellipsoids are drawn at the 30% probability level.

**Fig. 2.**
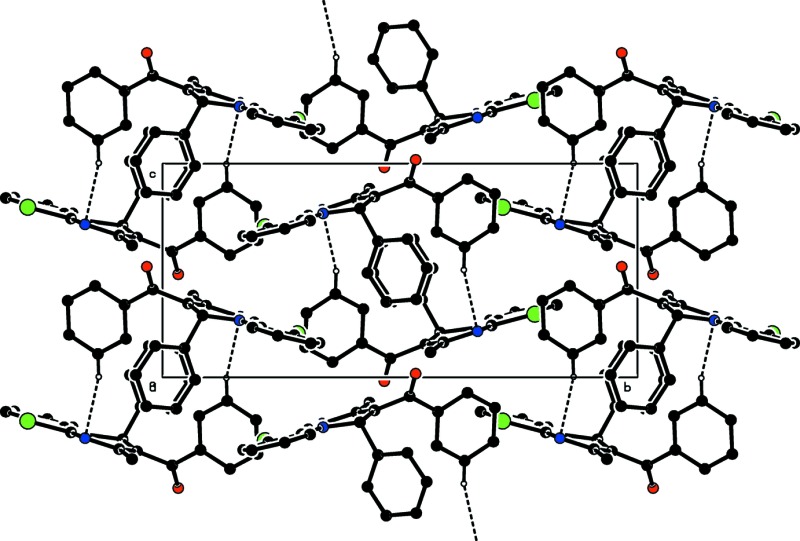
The crystal packing of the title compound viewed along the a axis. The hydrogen bonds are shown as dashed lines (see Table 1 for details).

. DOI: 10.1107/S2056989015006428/su5105fig1.tif
The mol­ecular structure of the title compound, with atom labelling. The displacement ellipsoids are drawn at the 30% probability level.

a . DOI: 10.1107/S2056989015006428/su5105fig2.tif
The crystal packing of the title compound viewed along the *a* axis. The hydrogen bonds are shown as dashed lines (see Table 1 for details).

CCDC reference: 1056199

Additional supporting information:  crystallographic information; 3D view; checkCIF report

## Figures and Tables

**Table 1:** Hydrogen-bond geometry (, )

*D*H*A*	*D*H	H*A*	*D* *A*	*D*H*A*
C27H27N1^i^	0.93	2.56	3.472(2)	166

Symmetry code: (i) 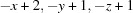.

## supplementary crystallographic information

### S1. Synthesis and crystallization

A mixture of benzaldehyde was treated with 1-phenyl butane 1-3-dione and 2-amino­benzo­thia­zole in the presence of ammonium acetate in ethanol. The mixture was gently warmed in a water bath at 353 K until the colour changed to yellow. It was kept aside overnight at room temperature. On completion of reaction (monitored by TLC) the solid obtained was separated and purified by column chromatography using hexane and ethyl acetate as eluent. The solid obtained was recrystallized using a 1:1 mixture of ethanol and THF giving yellow block-like crystals.

### S2. Structural commentary

Many pyrimidine derivatives show strong activity against bacteria, tumor and some viruses (Kumar *et al.*, 2012; Baraldi *et al.*, 2002; Nasr & Gineinah, 2002). Owing to the above said important properties of pyrimidine derivatives,the crystal structure determination of the title compound is carried out.

The molecular structure of the title compound is shown in Fig. 1. The pyrimidine ring [N1/C2/N19/C11—C13] has a envelope conformation with the maximum deviation of 0.1342 (17) Å shown by the flap atom C11. The attached phenyl ring [C15—C20] is twisted at an angle of 84.87 (8) ° with respect to pyrimidine ring mean plane. The thia­zolo group [S3/C2/N10/C4/C9] is also planar and fused with a benzene ring [C4—C9]. The phenyl­methanone ring [C23—C28] is almost perpendicular to the pyrimidine ring mean plane, with a dihedral angle between of 75.33 (9) Å.

In the crystal, molecules are linked via pairs of C—H···N hydrogen bonds forming inversion dimers (Table 1 and Fig. 2).

### S3. Refinement

Crystal data, data collection and structure refinement details are summarized in Table 2. H atoms were positioned geometrically (C—H = 0.93-0.98 Å) and allowed to ride on their parent atoms, with U_iso_(H) = 1.5U_eq_(C) for methyl H atoms and 1.2U_eq_(C) for other H atoms.

### Crystal data

**Table:**

C_24_H_18_N_2_OS	*F*(000) = 800
*M**_r_* = 382.46	*D*_x_ = 1.336 Mg m^−^^3^
Monoclinic, *P*2_1_/*c*	Cu *K*α radiation, λ = 1.54178 Å
Hall symbol: -P 2ybc	Cell parameters from 3047 reflections
*a* = 12.1894 (6) Å	θ = 4.8–64.4°
*b* = 18.6119 (8) Å	µ = 1.64 mm^−^^1^
*c* = 8.9370 (4) Å	*T* = 296 K
β = 110.360 (1)°	Block, yellow
*V* = 1900.85 (15) Å^3^	0.25 × 0.20 × 0.18 mm
*Z* = 4	

### Data collection

**Table:**

Bruker SMART APEXII CCD diffractometer	3090 independent reflections
Radiation source: fine-focus sealed tube	3047 reflections with *I* > 2σ(*I*)
Graphite monochromator	*R*_int_ = 0.036
ω and φ scans	θ_max_ = 64.4°, θ_min_ = 4.8°
Absorption correction: multi-scan (*SADABS*; Bruker, 2008)	*h* = −14→12
*T*_min_ = 0.709, *T*_max_ = 0.745	*k* = −21→19
11594 measured reflections	*l* = −10→10

### Refinement

**Table:**

Refinement on *F*^2^	Primary atom site location: structure-invariant direct methods
Least-squares matrix: full	Secondary atom site location: difference Fourier map
*R*[*F*^2^ > 2σ(*F*^2^)] = 0.040	Hydrogen site location: inferred from neighbouring sites
*wR*(*F*^2^) = 0.109	H-atom parameters constrained
*S* = 1.08	*w* = 1/[σ^2^(*F*_o_^2^) + (0.0592*P*)^2^ + 1.0365*P*] where *P* = (*F*_o_^2^ + 2*F*_c_^2^)/3
3090 reflections	(Δ/σ)_max_ = 0.001
254 parameters	Δρ_max_ = 0.28 e Å^−^^3^
0 restraints	Δρ_min_ = −0.33 e Å^−^^3^

### Special details

**Table:**

Geometry. All e.s.d.'s (except the e.s.d. in the dihedral angle between two l.s. planes) are estimated using the full covariance matrix. The cell e.s.d.'s are taken into account individually in the estimation of e.s.d.'s in distances, angles and torsion angles; correlations between e.s.d.'s in cell parameters are only used when they are defined by crystal symmetry. An approximate (isotropic) treatment of cell e.s.d.'s is used for estimating e.s.d.'s involving l.s. planes.
Refinement. Refinement of *F*^2^ against ALL reflections. The weighted *R*-factor *wR* and goodness of fit *S* are based on *F*^2^, conventional *R*-factors *R* are based on *F*, with *F* set to zero for negative *F*^2^. The threshold expression of *F*^2^ > σ(*F*^2^) is used only for calculating *R*-factors(gt) *etc*. and is not relevant to the choice of reflections for refinement. *R*-factors based on *F*^2^ are statistically about twice as large as those based on *F*, and *R*- factors based on ALL data will be even larger.

### Fractional atomic coordinates and isotropic or equivalent isotropic displacement parameters (Å^2^)

**Table:**

	*x*	*y*	*z*	*U*_iso_*/*U*_eq_	
S3	0.78290 (4)	0.78385 (2)	0.29579 (5)	0.02105 (16)	
O22	0.63518 (10)	0.46696 (6)	−0.01765 (14)	0.0218 (3)	
N10	0.67761 (11)	0.66193 (7)	0.23307 (16)	0.0153 (3)	
N1	0.86815 (12)	0.66330 (7)	0.21581 (17)	0.0185 (3)	
C23	0.81232 (14)	0.42002 (9)	0.1606 (2)	0.0176 (4)	
C15	0.66359 (13)	0.54627 (9)	0.36319 (19)	0.0158 (3)	
C21	0.72853 (14)	0.47969 (9)	0.08872 (18)	0.0159 (3)	
C9	0.59793 (14)	0.70747 (9)	0.26642 (19)	0.0168 (4)	
C13	0.85112 (14)	0.59148 (9)	0.16452 (19)	0.0166 (3)	
C20	0.59791 (15)	0.48448 (9)	0.3530 (2)	0.0199 (4)	
H20	0.5447	0.4701	0.2549	0.024*	
C8	0.48789 (15)	0.68936 (9)	0.2670 (2)	0.0212 (4)	
H8	0.4588	0.6430	0.2427	0.025*	
C12	0.75429 (14)	0.55374 (9)	0.15422 (18)	0.0156 (3)	
C11	0.65887 (13)	0.58425 (8)	0.20997 (19)	0.0151 (3)	
H11	0.5823	0.5756	0.1275	0.018*	
C24	0.81031 (16)	0.35791 (9)	0.0720 (2)	0.0239 (4)	
H24	0.7608	0.3552	−0.0339	0.029*	
C2	0.78084 (14)	0.69288 (9)	0.24274 (19)	0.0161 (3)	
C5	0.57494 (15)	0.83013 (9)	0.3392 (2)	0.0220 (4)	
H5	0.6034	0.8767	0.3622	0.026*	
C7	0.42230 (16)	0.74266 (10)	0.3051 (2)	0.0246 (4)	
H7	0.3483	0.7316	0.3068	0.030*	
C6	0.46508 (16)	0.81215 (10)	0.3406 (2)	0.0242 (4)	
H6	0.4195	0.8469	0.3656	0.029*	
C28	0.88470 (15)	0.42280 (10)	0.3196 (2)	0.0229 (4)	
H28	0.8869	0.4641	0.3792	0.027*	
C18	0.68805 (17)	0.46582 (10)	0.6348 (2)	0.0276 (4)	
H18	0.6972	0.4385	0.7255	0.033*	
C25	0.88206 (18)	0.30017 (10)	0.1416 (3)	0.0308 (4)	
H25	0.8824	0.2595	0.0814	0.037*	
C14	0.95013 (15)	0.56424 (10)	0.1177 (2)	0.0229 (4)	
H14A	0.9323	0.5167	0.0744	0.034*	
H14B	1.0204	0.5630	0.2099	0.034*	
H14C	0.9612	0.5955	0.0388	0.034*	
C4	0.64142 (15)	0.77729 (9)	0.30278 (19)	0.0183 (4)	
C16	0.74035 (15)	0.56846 (9)	0.5113 (2)	0.0219 (4)	
H16	0.7840	0.6102	0.5191	0.026*	
C17	0.75201 (16)	0.52884 (10)	0.6466 (2)	0.0257 (4)	
H17	0.8026	0.5442	0.7455	0.031*	
C27	0.95393 (16)	0.36400 (11)	0.3900 (2)	0.0297 (4)	
H27	1.0008	0.3656	0.4972	0.036*	
C26	0.95303 (17)	0.30319 (11)	0.3006 (3)	0.0307 (4)	
H26	1.0002	0.2642	0.3475	0.037*	
C19	0.61111 (17)	0.44390 (10)	0.4884 (2)	0.0269 (4)	
H19	0.5681	0.4019	0.4805	0.032*	

### Atomic displacement parameters (Å^2^)

**Table:**

	*U*^11^	*U*^22^	*U*^33^	*U*^12^	*U*^13^	*U*^23^
S3	0.0211 (3)	0.0137 (3)	0.0288 (3)	−0.00246 (15)	0.00924 (19)	−0.00270 (15)
O22	0.0202 (6)	0.0214 (6)	0.0200 (6)	0.0014 (5)	0.0022 (5)	−0.0039 (5)
N10	0.0169 (7)	0.0114 (7)	0.0184 (7)	−0.0003 (5)	0.0070 (5)	−0.0001 (5)
N1	0.0174 (7)	0.0168 (7)	0.0215 (7)	−0.0005 (5)	0.0071 (6)	0.0008 (6)
C23	0.0164 (8)	0.0159 (8)	0.0229 (8)	0.0006 (6)	0.0099 (7)	0.0014 (7)
C15	0.0152 (8)	0.0152 (8)	0.0188 (8)	0.0035 (6)	0.0080 (6)	−0.0007 (6)
C21	0.0175 (8)	0.0189 (9)	0.0135 (7)	0.0002 (6)	0.0082 (6)	0.0001 (6)
C9	0.0189 (8)	0.0161 (8)	0.0147 (8)	0.0033 (6)	0.0049 (6)	0.0001 (6)
C13	0.0166 (8)	0.0172 (8)	0.0150 (8)	0.0029 (6)	0.0041 (6)	0.0022 (6)
C20	0.0222 (8)	0.0180 (8)	0.0189 (8)	−0.0025 (7)	0.0064 (7)	−0.0014 (7)
C8	0.0217 (9)	0.0168 (9)	0.0265 (9)	−0.0008 (7)	0.0101 (7)	−0.0017 (7)
C12	0.0162 (8)	0.0161 (8)	0.0145 (8)	0.0030 (6)	0.0053 (6)	0.0014 (6)
C11	0.0150 (8)	0.0128 (8)	0.0168 (8)	−0.0002 (6)	0.0046 (6)	−0.0018 (6)
C24	0.0280 (9)	0.0201 (9)	0.0260 (9)	0.0026 (7)	0.0123 (7)	−0.0012 (7)
C2	0.0174 (8)	0.0151 (8)	0.0147 (8)	−0.0005 (6)	0.0041 (6)	0.0012 (6)
C5	0.0278 (9)	0.0147 (8)	0.0223 (9)	0.0020 (7)	0.0072 (7)	−0.0031 (7)
C7	0.0220 (9)	0.0246 (10)	0.0300 (9)	0.0031 (7)	0.0127 (7)	−0.0015 (7)
C6	0.0276 (9)	0.0203 (9)	0.0263 (9)	0.0071 (7)	0.0114 (7)	−0.0018 (7)
C28	0.0216 (9)	0.0210 (9)	0.0244 (9)	0.0016 (7)	0.0059 (7)	0.0008 (7)
C18	0.0384 (11)	0.0250 (10)	0.0222 (9)	0.0030 (8)	0.0140 (8)	0.0063 (7)
C25	0.0378 (11)	0.0180 (9)	0.0442 (12)	0.0065 (8)	0.0238 (9)	−0.0002 (8)
C14	0.0206 (9)	0.0223 (9)	0.0294 (9)	0.0014 (7)	0.0131 (7)	0.0012 (7)
C4	0.0204 (9)	0.0168 (8)	0.0164 (8)	0.0009 (6)	0.0048 (7)	0.0003 (6)
C16	0.0238 (9)	0.0201 (9)	0.0212 (9)	−0.0032 (7)	0.0070 (7)	−0.0025 (7)
C17	0.0294 (9)	0.0280 (10)	0.0172 (8)	0.0009 (8)	0.0050 (7)	−0.0016 (7)
C27	0.0247 (9)	0.0303 (10)	0.0311 (10)	0.0073 (8)	0.0060 (8)	0.0094 (8)
C26	0.0267 (10)	0.0237 (10)	0.0452 (12)	0.0110 (8)	0.0171 (9)	0.0126 (8)
C19	0.0353 (10)	0.0210 (9)	0.0271 (10)	−0.0060 (8)	0.0143 (8)	0.0021 (7)

### Geometric parameters (Å, º)

**Table:**

S3—C4	1.7515 (18)	C24—C25	1.389 (3)
S3—C2	1.7560 (16)	C24—H24	0.9300
O22—C21	1.225 (2)	C5—C4	1.383 (2)
N10—C2	1.359 (2)	C5—C6	1.385 (3)
N10—C9	1.397 (2)	C5—H5	0.9300
N10—C11	1.467 (2)	C7—C6	1.389 (3)
N1—C2	1.293 (2)	C7—H7	0.9300
N1—C13	1.405 (2)	C6—H6	0.9300
C23—C28	1.389 (2)	C28—C27	1.391 (3)
C23—C24	1.396 (2)	C28—H28	0.9300
C23—C21	1.494 (2)	C18—C19	1.380 (3)
C15—C20	1.386 (2)	C18—C17	1.392 (3)
C15—C16	1.391 (2)	C18—H18	0.9300
C15—C11	1.524 (2)	C25—C26	1.384 (3)
C21—C12	1.488 (2)	C25—H25	0.9300
C9—C8	1.385 (2)	C14—H14A	0.9600
C9—C4	1.398 (2)	C14—H14B	0.9600
C13—C12	1.349 (2)	C14—H14C	0.9600
C13—C14	1.497 (2)	C16—C17	1.381 (3)
C20—C19	1.388 (3)	C16—H16	0.9300
C20—H20	0.9300	C17—H17	0.9300
C8—C7	1.389 (3)	C27—C26	1.383 (3)
C8—H8	0.9300	C27—H27	0.9300
C12—C11	1.525 (2)	C26—H26	0.9300
C11—H11	0.9800	C19—H19	0.9300
			
C4—S3—C2	91.18 (8)	C4—C5—H5	120.7
C2—N10—C9	115.23 (13)	C6—C5—H5	120.7
C2—N10—C11	121.47 (13)	C8—C7—C6	121.25 (17)
C9—N10—C11	122.83 (13)	C8—C7—H7	119.4
C2—N1—C13	115.35 (14)	C6—C7—H7	119.4
C28—C23—C24	119.38 (16)	C5—C6—C7	120.67 (16)
C28—C23—C21	120.53 (15)	C5—C6—H6	119.7
C24—C23—C21	119.81 (15)	C7—C6—H6	119.7
C20—C15—C16	119.38 (16)	C23—C28—C27	120.18 (17)
C20—C15—C11	119.04 (14)	C23—C28—H28	119.9
C16—C15—C11	121.31 (15)	C27—C28—H28	119.9
O22—C21—C12	119.75 (14)	C19—C18—C17	119.92 (17)
O22—C21—C23	120.07 (15)	C19—C18—H18	120.0
C12—C21—C23	120.03 (13)	C17—C18—H18	120.0
C8—C9—N10	126.81 (15)	C26—C25—C24	119.97 (18)
C8—C9—C4	120.99 (15)	C26—C25—H25	120.0
N10—C9—C4	112.20 (14)	C24—C25—H25	120.0
C12—C13—N1	122.97 (15)	C13—C14—H14A	109.5
C12—C13—C14	125.18 (15)	C13—C14—H14B	109.5
N1—C13—C14	111.81 (14)	H14A—C14—H14B	109.5
C15—C20—C19	120.36 (16)	C13—C14—H14C	109.5
C15—C20—H20	119.8	H14A—C14—H14C	109.5
C19—C20—H20	119.8	H14B—C14—H14C	109.5
C9—C8—C7	117.90 (16)	C5—C4—C9	120.64 (16)
C9—C8—H8	121.1	C5—C4—S3	128.57 (14)
C7—C8—H8	121.1	C9—C4—S3	110.79 (12)
C13—C12—C21	124.69 (15)	C17—C16—C15	120.32 (16)
C13—C12—C11	122.17 (15)	C17—C16—H16	119.8
C21—C12—C11	113.13 (13)	C15—C16—H16	119.8
N10—C11—C15	112.04 (13)	C16—C17—C18	119.97 (16)
N10—C11—C12	108.29 (13)	C16—C17—H17	120.0
C15—C11—C12	109.03 (12)	C18—C17—H17	120.0
N10—C11—H11	109.1	C26—C27—C28	120.05 (18)
C15—C11—H11	109.1	C26—C27—H27	120.0
C12—C11—H11	109.1	C28—C27—H27	120.0
C25—C24—C23	120.18 (17)	C27—C26—C25	120.19 (17)
C25—C24—H24	119.9	C27—C26—H26	119.9
C23—C24—H24	119.9	C25—C26—H26	119.9
N1—C2—N10	127.70 (15)	C18—C19—C20	120.02 (17)
N1—C2—S3	121.70 (13)	C18—C19—H19	120.0
N10—C2—S3	110.59 (12)	C20—C19—H19	120.0
C4—C5—C6	118.55 (16)		

### Hydrogen-bond geometry (Å, º)

**Table:**

*D*—H···*A*	*D*—H	H···*A*	*D*···*A*	*D*—H···*A*
C27—H27···N1^i^	0.93	2.56	3.472 (2)	166

Symmetry code: (i) −*x*+2, −*y*+1, −*z*+1.
