# Supplementary figures and images for: Crystal structure of (2-methyl-4-phenyl-4H-benzo[4,5]thia­zolo[3,2-a]pyrimidin-3-yl)(phen­yl)methanone
Source: Acta Crystallogr E Crystallogr Commun. 2015 Apr 2;71(Pt 5):o276–7. doi: 10.1107/S2056989015006428 (PMC4420141; doi:10.1107/S2056989015006428)

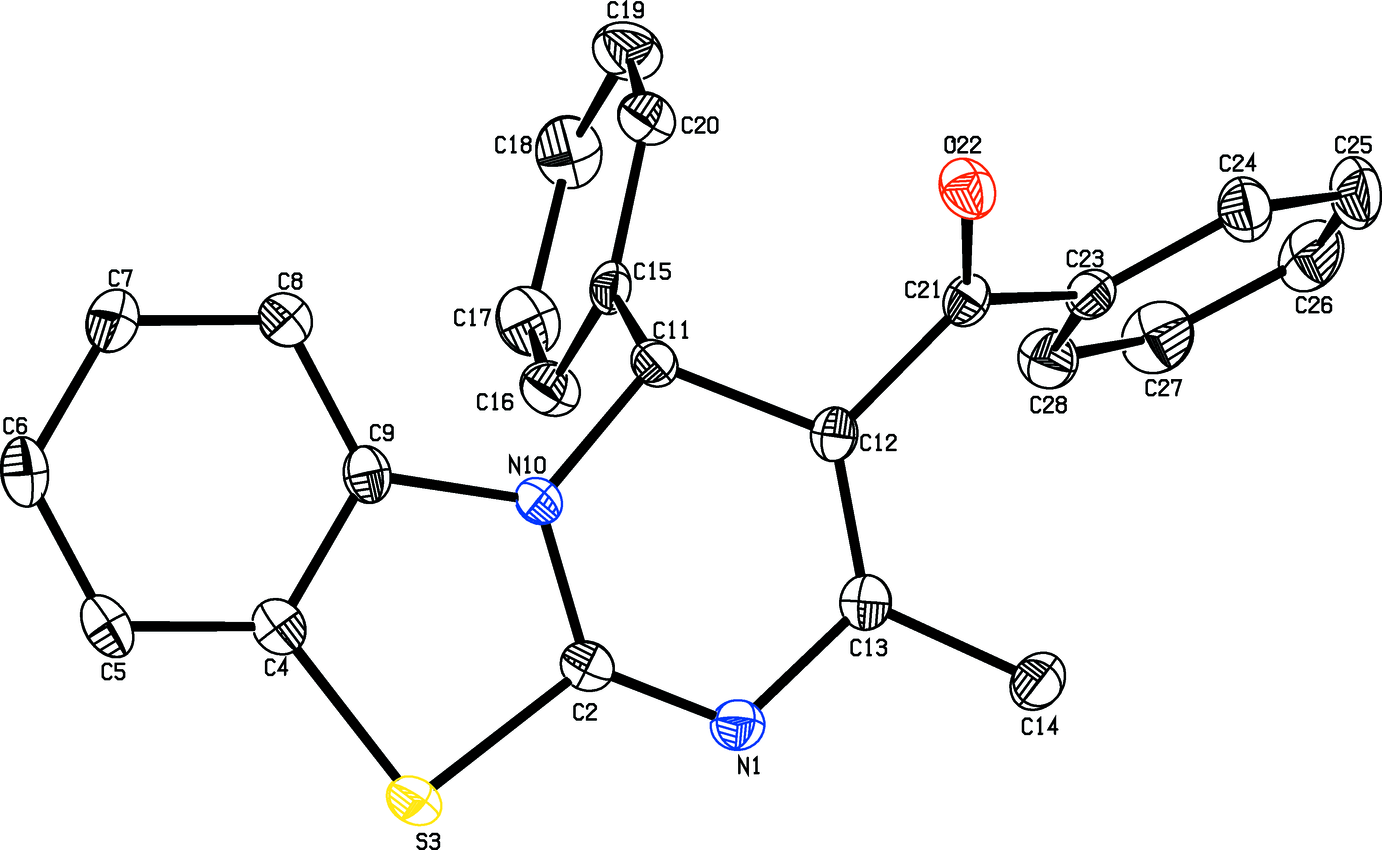

Supplement: Supplementary file 4 [file e-71-0o276-fig1.tif]

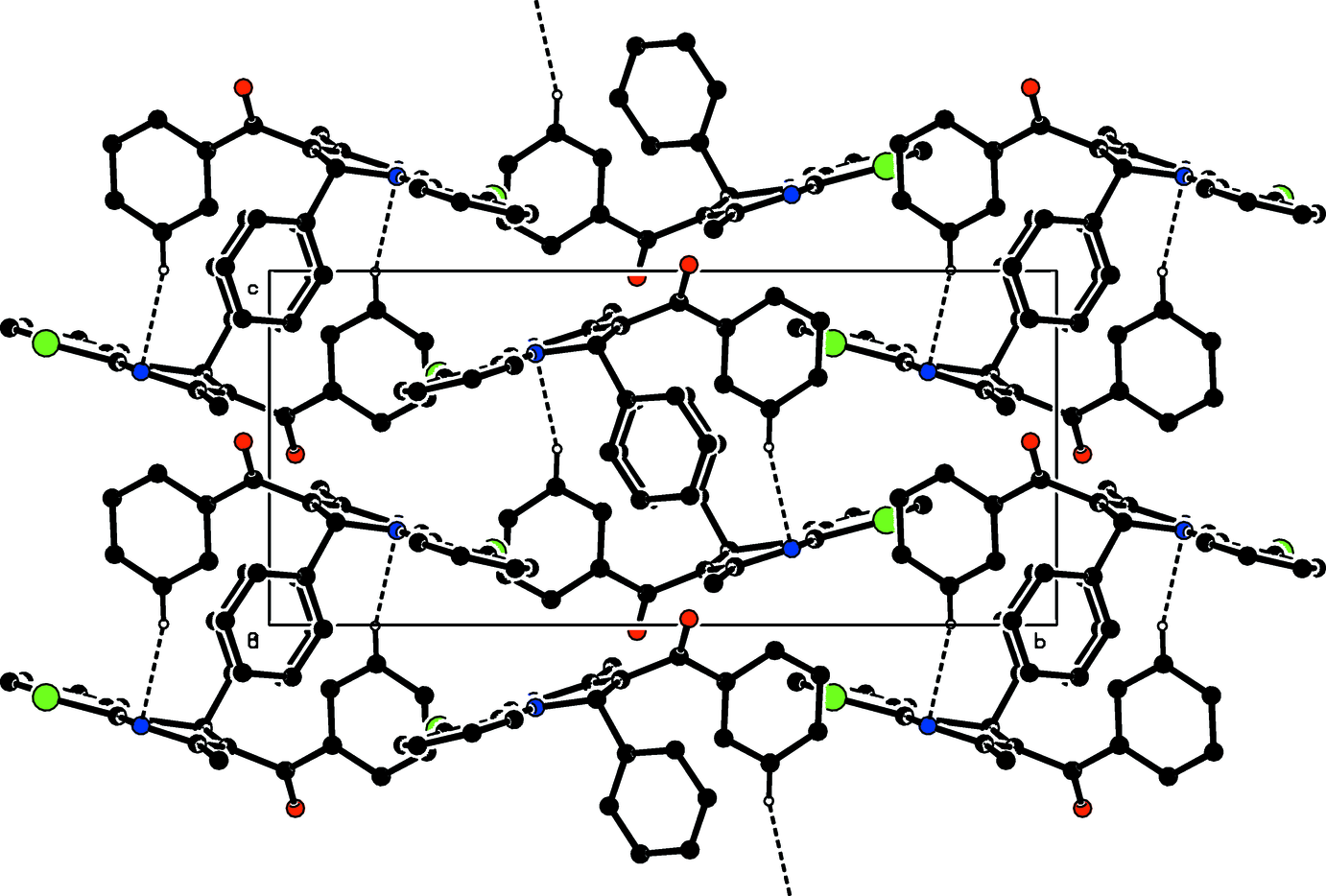

Supplement: Supplementary file 5 [file e-71-0o276-fig2.tif]
